# Profiling of circulating chromosome 21‐encoded microRNAs, miR‐155, and let‐7c, in down syndrome

**DOI:** 10.1002/mgg3.1938

**Published:** 2022-04-12

**Authors:** Jesús Manuel Pérez‐Villarreal, Katia Aviña‐Padilla, Evangelina Beltrán‐López, Alma Marlene Guadrón‐Llanos, Esther López‐Bayghen, Javier Magaña‐Gómez, Marco Antonio Meraz‐Ríos, Alfredo Varela‐Echavarría, Carla Angulo‐Rojo

**Affiliations:** ^1^ Laboratorio de Neurociencias, Centro de Investigación Aplicada a la Salud Pública (CIASaP), Facultad de Medicina Universidad Autónoma de Sinaloa Culiacán Mexico; ^2^ Maestría en Ciencias Biomédicas, Facultad de Ciencias Químico‐Biológicas Universidad Autónoma de Sinaloa Culiacán Mexico; ^3^ Laboratorio de Nutrición Molecular, Escuela de Nutrición y Gastronomía Universidad Autónoma de Sinaloa Culiacán Mexico; ^4^ Instituto de Neurobiología Universidad Nacional Autónoma de México Querétaro Mexico; ^5^ Laboratorio de Bioinformática y de Redes Complejas Centro de Investigación y de Estudios Avanzados del Instituto Politécnico Nacional (CINVESTAV‐IRAPUATO) Mexico; ^6^ Laboratorio Edificio Central, Facultad de Ciencias Químico‐Biológicas Universidad Autónoma de Sinaloa Culiacán Mexico; ^7^ Laboratorio de Diabetes y comorbilidades, Centro de Investigación Aplicada a la Salud Pública (CIASaP), Facultad de Medicina Universidad Autónoma de Sinaloa Culiacán Mexico; ^8^ Departamento de Toxicología Centro de Investigación y de Estudios Avanzados del Instituto Politécnico Nacional (CINVESTAV‐IPN) México City Mexico; ^9^ Departamento de Biomedicina Molecular Centro de Investigación y de Estudios Avanzados del Instituto Politécnico Nacional (CINVESTAV‐IPN) México City Mexico; ^10^ Centro de Investigación y Docencia en Ciencias de la Salud (CIDOCS) Universidad Autónoma de Sinaloa Culiacán Mexico

**Keywords:** Alzheimer's disease, down syndrome, dyslipidemias, microRNAs, obesity

## Abstract

**Background:**

Down syndrome (DS) is the most common chromosomal survival aneuploidy. The increase in DS life expectancy further heightens the risk of dementia, principally early‐onset Alzheimer's disease (AD). AD risk in DS is higher, considering that this population may also develop metabolic diseases such as obesity, dyslipidemias, and diabetes mellitus. The extra genetic material that characterizes DS causes an imbalance in the genetic dosage, including over‐expression of AD's key pathophysiological molecules and the gene expression regulators, the microRNAs (miRNAs). Two miRNAs, chromosome 21‐encoded, miR‐155, and let‐7c, are associated with cognitive impairment and dementia in adults; but, expression dynamics and relationship with clinical variables during the DS's lifespan had remained hitherto unexplored.

**Methods:**

The anthropometric, clinical, biochemical, and profile expression of circulating miR‐155 and let‐7c were analyzed in a population of 52 control and 50 DS subjects divided into the young group (Aged ≤20 years) and the adult group (Aged ≥21 years).

**Results:**

The expression changes for miR‐155 were not significant; nevertheless, a negative correlation with HDL‐Cholesterol concentrations was observed. Notably, let‐7c was over‐expressed in DS from young and old ages.

**Conclusion:**

Overall, our results suggest that let‐7c plays a role from the early stages of DS's cognitive impairment while overexpression of miR‐155 may be related to lipid metabolism changes. Further studies of both miRNAs will shed light on their potential as therapeutic targets to prevent or delay DS's cognitive impairment.

## Graphical abstract

Extra chromosome 21 may imbalance key pathological miRNAs, miR‐155, and let7c. Overexpression of miR‐155 may be related to lipid metabolism changes, meanwhile, let‐7c may play a role from the early stages of Down syndrome cognitive impairment.

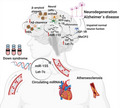

## 1 INTRODUCTION

Down syndrome (DS), or trisomy 21, is the most common survivable chromosomal aneuploidy, occurring at a rate that ranges between 6 and 13 in 10,000 births (de Graaf et al., 2017; Sierra Romero et al., 2014). The prevalence of DS increased in recent decades as the life expectancy of people with this condition has expanded to 60 years and beyond (Saghazadeh et al., 2017), leading to a primarily understudied population in the aging process. Furthermore, adults with DS are at higher risk for early onset Alzheimer's disease (AD), the most common form of dementia (Antonarakis et al., 2004). It was recently reported that adults aged 40–54 years have a 40% probability of an incident dementia claim, which increases to 67% for those 55 years and older (Rubenstein et al., 2020). Moreover, DS individuals have an increased risk of overweight, obesity, and dyslipidemias, which are also risk factors for AD (Bertapelli et al., 2016). Hence, there is a critical need to identify biomarkers to track neuropathology and cognitive decline in DS people to prevent it and provide proper medical care to this population.

Most of the features of DS relate to over‐expression of the genes located on chromosome 21, particularly those of the Down Syndrome Critical Region (DSCR), leading to imbalanced interactions with other disomic genes (Asim et al., 2015). In DS individuals, the increased risk of early‐onset Alzheimer's disease is related to the trisomic dosage of the genes for amyloid precursor protein (*APP*, OMIM #104760) and β secretase 2 (*BACE2*, OMIM #605668), essential proteins for amyloid‐beta (Aβ) peptide production (Ballard et al., 2016). Deposits in the brain of Aβ peptide, along with neurofibrillary tangles caused by hyperphosphorylation and accumulation of Tau protein, are the pathological hallmarks of AD (Scheltens et al., 2016).

Furthermore, other genes for early molecular players in the pathophysiology of DS and AD such as microRNAs (miRNAs), are also located on chromosome 21 (Antonarakis, 2017). Their expression during lifespan, however, remains thus far uncharacterized.

miRNAs are small non‐coding strands of RNA (18–25 nucleotides), which regulate post‐transcriptionally almost 60% of all gene expression via inhibition of translation or direct mRNA target degradation (Friedman et al., 2009). These small regulatory RNAs often have tissue‐specific expression, are detectable in peripheral blood, and may act as endocrine effectors on target cells. Hence, they have been proposed as circulating biomarkers for neurodegeneration.

Human chromosome 21 has more than 400 genes, including five miRNAs (miR‐99a, let‐7c, miR‐125b‐2, miR‐155, and miR‐802) whose expression leads to haploinsufficiency for their mRNA target (Elton et al., 2010). miRDB database analysis (http://mirdb.org/index.html) (Chen & Wang, 2019) predicted that miR‐99a is a potential interactor of 47 mRNA targets, let‐7c of 990, miR‐125b‐2 of 585, miR‐155 of 701, and miR‐802 of 631. It follows then that dysregulation of these miRNAs may play a role in the cognitive dysfunction and early‐onset AD in individuals with DS.

Among those miRNAs, we focused on let‐7c and miR‐155, which are critically related to neurodegeneration and play a crucial role in DS pathogenesis of dementia (McGowan et al., 2018; Tili et al., 2018). The extra copy of miR‐155 may contribute to neuroinflammation in AD related to NF‐kB signaling, T cell activation, and infiltration in the hippocampal brain parenchyma (Tili et al., 2018). Moreover, miR‐155 is co‐expressed with hyperphosphorylated Tau (Ballard et al., 2016; Tili et al., 2018), and over‐expression of let‐7c leads to impaired neuronal morphologic development, synapse formation, and synaptic strength, as well as a marked reduction of neuronal excitability (Chen et al., 2018; McGowan et al., 2018). In addition to the known interactors of let‐7c and miR‐155, there are unexplored mRNA targets involved in processes linked both to DS and AD pathology. For example, DS people are at risk of dyslipidemia characterized by high levels of triglycerides and low‐density lipoprotein cholesterol (LDL‐Col) and low levels of high‐density lipoprotein Cholesterol (HDL‐Col) (Adelekan et al., 2012; Buonuomo et al., 2016; de la Piedra et al., 2017; Tenneti et al., 2017). In turn, dyslipidemia in these individuals increases the risk of AD, as HDL‐Col is required for the Aβ peptide clearance (Kuo et al., 1998; Robert et al., 2017).

Therefore, in the present work, we focused on profiling the expression of circulating miR‐155 and let‐7c miRNAs in the young and adult DS population and exploring its possible correlations with AD risk factors present in DS, such as the lipid profile.

## 2 MATERIALS AND METHODS

### 2.1 Study design

A cross‐sectional study was performed with 50 participants with DS and 52 healthy controls. Individuals were selected at Down Syndrome Health Fairs organized by the Autonomous University of Sinaloa.

DS individuals and controls were each grouped in 20‐year‐old or younger (Group A) and 21‐year‐old or older (Group B), according to the growth standards for children with DS by the Disease Control and Prevention Center (CDC).

### 2.2 Patients

The study's inclusion criteria were age over 8 years and a DS diagnosis by clinical and genetic criteria. Sex and age‐matched control participants were used as a reference. Exclusion criteria were the presence of congenital heart disease not treated with surgery, severe sensory impairment, leukemia, and diagnosis of diabetes mellitus or metabolic syndrome.

### 2.3 Anthropometric measurements

Anthropometric measurements were carried out following the procedures of the International Society for the Advancement of Kinanthropometry (ISAK) during patient examinations. Bodyweight was measured on subjects wearing light clothes using an electronic bascule (Tanita HS‐302) and height using a stadiometer (SECA 213). Body mass index (BMI) was calculated, and individuals were classified according to the World Health Organization's BMI/Age tables (WHO).

### 2.4 Biochemical analysis

After an overnight fast, blood samples were obtained from the antecubital vein and collected into two non‐additive tubes. They were incubated for 30 min at room temperature and spun at 3,000*g* at 4°C for 10 min. Serum samples were collected and stored at −80°C. Serum lipids (total cholesterol and HDL‐cholesterol), triglycerides, and plasma glucose were measured using commercial enzymatic kits (HUMAN Diagnostics Worldwide; Wiesbaden, Germany). LDL‐cholesterol was calculated using a modified Friedewald equation (Friedewald et al., 1972). For the classification of biochemical parameters, we used the cut‐off points of the International Diabetes Federation (IDF) and the National Cholesterol Education Program (NCEP) for children, adolescents, and adults (NIH and NHLBI, 2001; Zimmet et al., 2007). The cutoff point is ≥200 mg/dl for total cholesterol, ≥130 mg/dl for LDL‐cholesterol, ≥150 mg/dl for triglycerides, and ≥ 100 mg/dl for glucose, applicable for all ages. In the case of HDL‐cholesterol <40 mg/dl (8–16 years old, male/female) and <40/50 mg/dl (people >16 years old, male/female).

### 2.5 RNA isolation

According to the manufacturer's protocol; total miRNA purification from 200 μl of serum was performed with the miRNeasy‐serum/plasma extraction kit (Qiagen, Hilden, Germany). Samples were eluted with RNase‐free water for subsequent cDNA synthesis and stored at −80°C.

### 2.6 Reverse transcription (RT) and real‐time PCR

Total RNA was reverse transcribed using a miScript II RT Kit (Qiagen) in a StepOne Plus (Thermo Fisher Scientific) according to the manufacturer's protocol and using equal volumes for all samples.

Quantification of let‐7c (cat. no. 00003129) and miR‐155 (cat. no. 00031486) mature miRNAs were determined using the miScript SYBR Green PCR kit (Qiagen) and SNORD68 (cat. no. 00033712) as normalizer (Abd‐El‐Fattah et al., 2013; Kumar et al., 2018). PCR reactions were each performed in a final volume of 15 μl in the StepOne Plus thermocycler (Applied Biosystems). The Melting Curve was performed to observe the specificity of reactions. Relative quantification was obtained using the Pfaffl method (Pfaffl, 2001), which describes the use of reaction efficiencies obtained using LinReg PCR software (Ruijter et al., 2009).

### 2.7 In silico prediction of miRNAs and their target genes

For predicting miR‐155 and let‐7c gene targets, miRDB (http://www.mirdb.org) public database was used (Agarwal et al., 2015; Chen & Wang, 2019). To determine the biological processes under each miRNA regulation, we performed network enrichment analysis using Metascape (Zhou et al., 2019) (http://metascape.org/). Then, we selected GO terms in miR155 targets related to lipid metabolism. Finally, we used the Meta‐analysis workflow to compare miR155 and let‐7c targets and to identify unique and shared biological pathways in which they are involved.

### 2.8 Statistical analysis

Statistical tests were performed using SPSS v.22. Data were examined for normality based on skewness and kurtosis before analysis. Descriptive statistics of clinical characteristics between groups were represented as means ± standard deviation. Mann–Whitney U and Student's *t*‐test were used to examine the statistical difference in clinical parameters between healthy controls subjects and DS patients. ANOVA Kruskal‐Wallis test for differences between groups with respect to miRNA expression was assessed. Spearman correlation coefficients determined correlations between fold changes of miRNA levels and changes in biochemical characteristics. Statistical significance was set at *p* < 0.05.

## 3 RESULTS

### 3.1 Anthropometry and biochemical parameters

The circulating profile expression of chromosome 21‐encoded miRNAs miR‐155 and let‐7c may be impacted by risk factors as clinical variables related to metabolism. To better characterize these variables, we analyzed anthropometry and biochemical parameters in control and DS populations. A total of 50 individuals with Down syndrome and 52 healthy controls from 8 to 52 years of age were characterized and divided into two groups: A (A Control and A DS, aged ≤20 years) and B (B Control and B DS, aged ≥21 years). All individuals were matched by sex and age. The mean age was 10.9 ± 2.4 and 11.3 ± 2.7 years for A Control and A DS groups, respectively; and 28.3 ± 11 and 28.5 ± 11 years for B Control and B DS groups (Table 1), respectively, with no significant difference between them, which indicates homogeneity of age‐divided groups. For bodyweight, there was no significant difference between A groups (Control 41.4 ± 12.5 and DS 37.9 ± 13.2 kg) and B groups (Control 63.3 ± 10.3 kg and DS 64.3 ± 12.7 kg) (Table 1) (all results are presented in this order heretofore).

**TABLE 1:** Participant characteristics and biochemical parameters

Clinical characteristics	Control A	DS A	*p*‐value	Control B	DS B	*p*‐value
Age (years)	10.9 ± 2.4	11.3 ± 2.7	NS	28.3 ± 11	28.5 ± 11	NS
Bodyweight (kg)	41.4 ± 12.5	37.9 ± 13.2	NS	63.3 ± 10.3	64.3 ± 12.7	NS
Height (m)	1.45 ± 0.11	1.3 ± 0.12	0.0001	1.6 ± 0.07	1.5 ± 0.08	0.0001
BMI (kg/m^2^)	19.2 ± 3.3	20.5 ± 4.3	NS	22.9 ± 2.6	28.8 ± 6.0	0.006
Glucose (mg/dl)	86.4 ± 11.1	87.4 ± 10.4	NS	91.08 ± 5.1	91 ± 13.6	NS
Cholesterol (mg/dl)	118.7 ± 30.2	181.2 ± 44.6	0.0001	157.4 ± 60.6	171.7 ± 54.2	NS
HDL (mg/dl)	56.8 ± 15.4	46.7 ± 13.17	0.003	68.8 ± 13.2	42.5 ± 9.1	0.0001
Triglycerides (mg/dl)	75.6 ± 43.3	139.1 ± 44.5	0.0001	80.3 ± 24.5	152.7 ± 83.5	0.011
LDL (mg/dl)	109.6 ± 43.1	106.4 ± 37.6	NS	72.8 ± 55.1	115 ± 28.07	0.029

*Note*: Data are presented as mean ± SD.

Abbreviations: BMI, body mass index; HDL, high‐density lipoprotein cholesterol; LDL, low‐density lipoprotein cholesterol; NS, non‐significative.

Nevertheless, we observed a significant difference in height between A groups (Control 1.45 ± 0.11 m and DS 1.3 ± 0.12 m, *p =* 0.001) and B groups (Control 1.6 ± 0.07 m and DS 1.5 ± 0.08 m, *p* = 0.001). Moreover, a significant difference in BMI was found between B groups (Control 22.9 ± 2.6 and DS 28.8 ± 6.0 m/kg^2^, *p* = 0.006), while no difference was observed between A groups (Table 1). Altogether, these results indicate that the risk of overweight in DS individuals is higher at older stages, in accordance with previous reports (Basil et al., 2016; Bertapelli et al., 2016).

Biochemical analysis revealed a significant difference between Control and DS A groups in total cholesterol (118.7 ± 30.2 and 81.2 ± 44.65 mg/dl, *p* = 0.0001), HDL (56.82 ± 15.4 and 46.7 ± 13.17 mg/dl, *p =* 0.003) and triglycerides (75.65 ± 43.33 and 139.1 ± 44.53 mg/dl, *p* = 0.0001), but not for LDL and glucose levels (Table 1). Similarly, for B groups we observed differences in HDL (68.8 ± 13.2 and 42.5 ± 9.1 mg/dl, *p* = 0.0001), triglycerides (80.3 ± 24.5 and 152.7 ± 83.5 mg/dl, *p* = 0.011) and LDL (72.8 ± 55.15 and 115 ± 28.07 mg/dl, *p* = 0.029), but not for total cholesterol and glucose (Table 1). Overall, these results show alterations in lipid metabolism parameters due to the genetic background of DS.

### 3.2 Serum miRNAs expression

**FIGURE 1:**
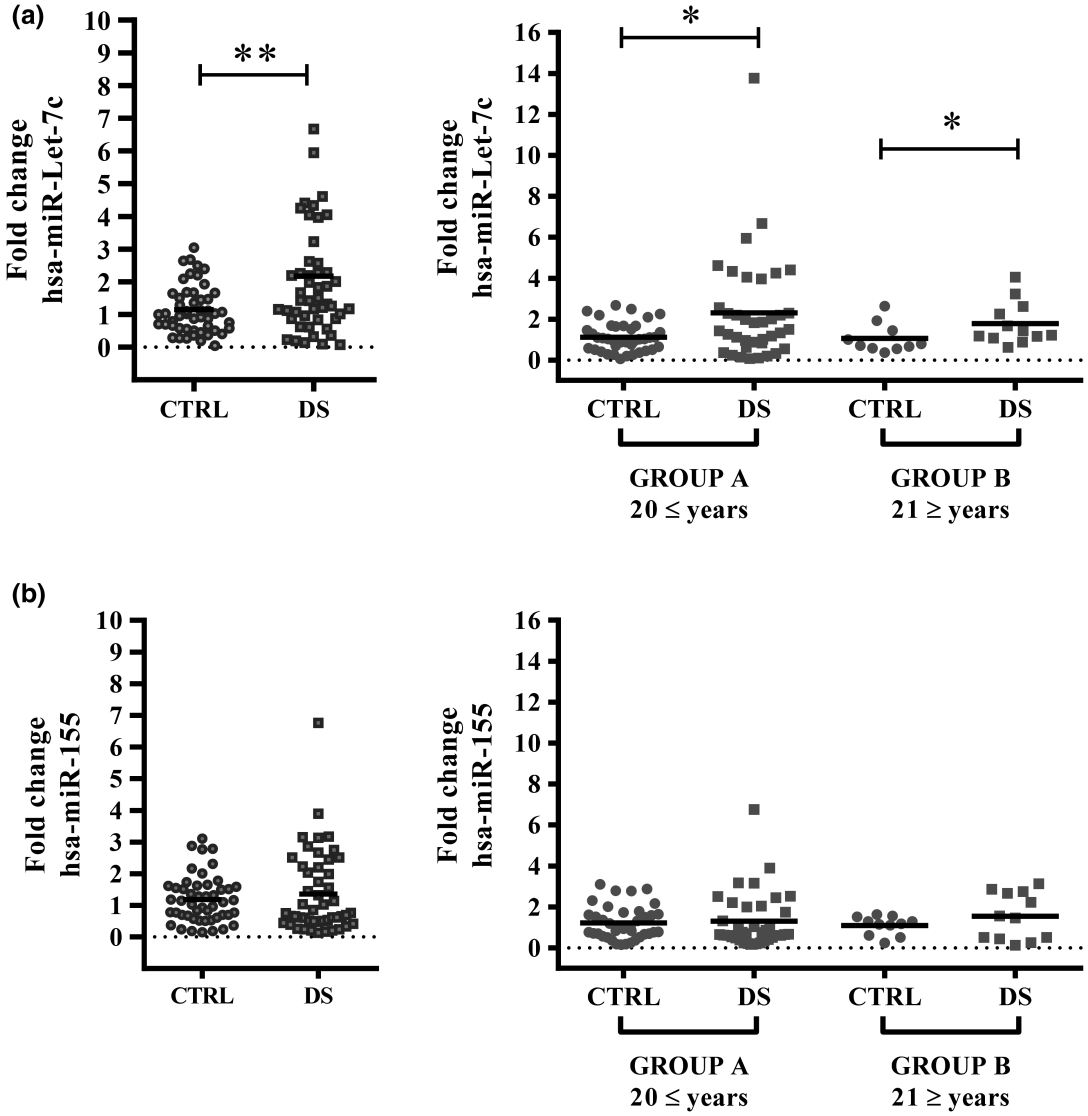
The miRNA let‐7c is up‐regulated in DS but not miR‐155. Expression of miRNAs let‐7 c (a) and miR‐155 (b) was measured by qRT‐PCR in DS people and referenced to control individuals. For each miRNA, total and age‐divided population were done. Data are plotted as relative value for each individual and means for each group statistically analyzed by Mann–Whitney U test (*p < 0.05, **p < 0.01). DS: Down syndrome; group a: ≤20 years old; group B: ≥21 years old

Analysis by qRT‐PCR of let‐7c expression revealed a 2.2‐fold increase in DS relative to the control group (Figure 1a) for the overall population. Moreover, when analyzed according to age, we observed a similar over‐expression of let‐7c in DS groups compared to their respective control groups in the young and adult populations (DS A: 2.3‐fold and DS B: 1.8‐fold) (Figure 1a). Nevertheless, some DS overexpresses miR‐155, no significant differences were detected for miR‐155 in both analyses (Figure 1b).

We then analyzed differences in the miRNA expression levels classifying the DS population according to normal or altered clinical parameters related to metabolism, as described in materials and methods. When focusing on lipid parameter alterations, we did not find differences in expression levels of miR‐155 between normal or altered lipid parameter subgroups (Table 2). Interestingly, the most remarkable change detected in the expression of let‐7c was among the Low‐HDL DS population (2.9‐fold) and this effect was lower in the normal‐HDL DS group (1.7‐fold) (Table 2).

**TABLE 2:** miRNAs expression levels according to lipid concentration, in down syndrome groups

miRNAs	DS Normal‐HDL	DS low‐HDL	*p*‐value
Let‐7c	1.7	2.9	0.0009
miR‐155	1	1.9	NS

	DS normal‐TG	DS high‐TG	
Let‐7c	2.4	1.6	0.0007
miR‐155	1.4	1.2	NS

	DS Normal‐cholesterol	DS high‐cholesterol	
Let‐7c	2.2	2.0	0.02
miR‐155	1.3	1.3	NS

	DS Normal‐LDL	DS High‐LDL	
Let‐7c	2.3	1.8	0.005
miR‐155	1.3	1.3	NS

*Note*: Data are presented as mean.

Abbreviations: HDL, High‐density lipoprotein cholesterol; LDL, Low‐density lipoprotein cholesterol; NS, non‐significative; TG, triglycerides.

### 3.3 Correlation between miRNA expression and biochemical parameters

Dyslipidemias are a risk factor for chronic complications in DS and may increase the risk of Alzheimer's disease. Since lipid concentrations have been linked to cognitive impairment, patients' biochemical parameters were correlated with expression values of miRNAs let‐7c and miR‐155 (Table 3). We did not find a correlation for let‐7c levels with any lipid or glucose parameter (Table 3). However, we observed a negative correlation between the HDL levels and the increase in the expression of miR‐155 (*p* = 0.004, r = −0.395) (Table 3). Interestingly, an overall landscape showed that DS individuals have let‐7c miRNA expression up‐regulated, especially in individuals with low‐HDL (Tables 2 and 3). Meanwhile, miR‐155 levels appear up‐regulated only when HDL levels are far below normal (Tables 2 and 3). Hence, the results suggest that only miR‐155 mRNA targets relate to the regulation of lipid metabolism.

**TABLE 3:** Bivariate correlation of let‐7c and miR‐155 levels with changes in clinical characteristics

	Let‐7c		miR‐155	
	*r*	*p*	*r*	*p*
Glucose	−0.217	0.134	0.004	0.977
Triglycerides	−0.132	0.363	−0.181	0.211
Cholesterol	−0.061	0.675	−0.086	0.553
LDL‐C	0.123	0.397	0.011	0.935
HDL‐C	−0.217	0.133	−0.395	0.004

Abbreviations: HDL, high‐density lipoprotein cholesterol; LDL, low‐density lipoprotein cholesterol.

### 3.4 Functional enrichment of gene targets of miR‐155 and let‐7c

**FIGURE 2:**
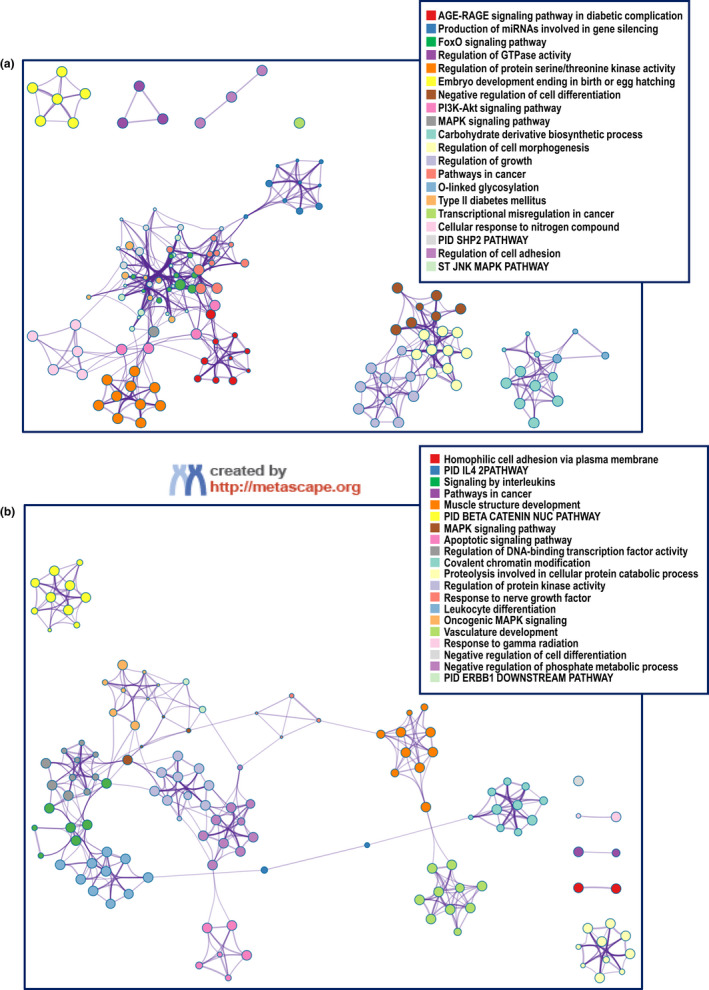
Networks of enriched ontology clusters for let‐7c (a) and miR‐155 (b) miRNAs regulated targets. GO: BP, gene ontology: biological processes terms

**FIGURE 3:**
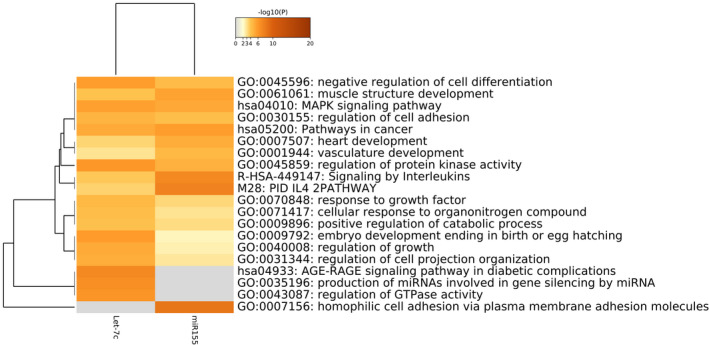
Heatmap of GO: BP enriched terms across miR155 and let‐7c target genes colored by *p*‐values. GO: BP, gene ontology: Biological processes terms

To gain insight into the role of miR‐155 and let‐7c targets that could be differentially affected in DS comorbidities, we performed an in‐silico analysis to find enriched biological pathways. miR‐155 and let‐7c targets were determined using the miRDB (http://www.mirdb.org) public database. Clustering networks of the top 20 enriched GO: BP (Gene Ontology: Biological Processes) terms for miR‐155 and let‐7c targets are shown in Figure 2. In‐silico prediction analysis showed that miR‐155 has 701 mRNA targets, while let‐7c could be regulating 990 genes. Comparative analysis of their targets showed that only 52 genes could be regulated by both miRNAs (Figure 3 and Figure S1). Despite the discrepancy in genetic regulation, both miRNAs are linked at the biological pathway level (Figure S1). It is noteworthy that enrichment analysis of biological processes revealed that the highest *p*‐value enriched GO: BP terms are unique pathways for each miRNA (Figures 2 and 3).

Bioinformatic analyses of let‐7c gene targets revealed enrichment of the signaling pathways associated with diseases, particularly to type 2 diabetes, such as the AGE/RAGE pathway and the carbohydrate derivate biosynthetic process (Figure 2a). A possible mechanism of let‐7c genetic regulation is the signaling cascade events of the IGF‐1/IGF‐1R pathway, which induces the phosphorylation and inhibition of forkhead (FoxO) transcription factors. The FoxO signaling pathway, highly enriched among let‐7c targets, is involved in the regulation of both survival and apoptotic genes by inducing the synthesis of the ligand of the apoptosis stimulating fragment (FasL) and the death mediator that interacts with BcL‐2 (Bim) (Figure 2a). Overall, these results suggest a function for let‐7c in cell survival and carbohydrate metabolism linked to the molecular etiology of neurodevelopmental disorders, such as DS.

Among the miR‐155 unique gene targets, we observed a high enrichment for a group of homophilic adhesion proteins via plasma membrane adhesion composed mostly of clustered alfa protocadherins (Pcdhs) and other non‐clustered members (Figure 2b). Also, miR‐155 is related to inflammatory pathways such as interleukin signaling and oncogenic signaling. Due to among the top 20 enriched GO: BP terms for miR‐155, no highly enriched terms associated with lipid metabolism were found, we performed further analysis for lipid‐linked biological processes from all the significantly enriched terms. We identified 60 miR155‐target genes involved in those lipid metabolism processes, with enrichment in GO: BP associated with cellular response to lipid, inositol lipid‐mediated signaling, long‐chain fatty acid import, positive regulation of lipid localization, and fat cell differentiation (−log10 ranking from −3.499 to −2.058) (Table 4 and Figure S2). Furthermore, miR‐155 mRNAs targets are implicated in the Reactome pathway R‐HSA‐400206: Regulation of lipid metabolism by PPAR alpha (−log10–2.51) (Table 4). The latest deep bioinformatics analysis applied to let‐7c did not find mRNA targets related to lipid metabolism (data not shown).

**TABLE 4:** Selected pathways and GO terms of miR155 targets associated with lipid metabolism

GO	Category	Parental GO	Description	Genes	LogP	Enrichment	Z‐score
GO biological processes							
GO:0045444	GO Biological Processes	19_GO:0032502 developmental process	Fat cell differentiation	ARNTL, CEBPB, GATA3, HNRNPU, ENPP1, RORA, RREB1, SORT1, TCF7L2, WNT5A, PIAS1, SOCS1, MAFB, TRIM32, ZADH2, PTPRQ	−3.499	2.715	4.234
GO:0045599	GO Biological Processes	19_GO:0048519 negative regulation of biological process	Negative regulation of fat cell differentiation	ARNTL, GATA3, ENPP1, RORA, SORT1, WNT5A, ZADH2	−3.476	5.289	5.002
GO:0045598	GO Biological Processes	19_GO:0032502 developmental process	Regulation of fat cell differentiation	ARNTL, CEBPB, GATA3, HNRNPU, ENPP1, RORA, RREB1, SORT1, WNT5A, ZADH2, PTPRQ	−3.019	3.080	3.991
GO:0071396	GO Biological Processes	19_GO:0050896 response to stimulus	Cellular response to lipid	ABL2, ARNTL, ARRB2, CD36, CEBPB, ABCC2, DEFA5, HNRNPU, IRF8, NR3C2, MYB, NKX3‐1, PDK4, PTN, RELA, RORA, SMARCA4, SOX9, SOX10, WNT5A, PIAS2, PDCD4, CD274, STRN3, KDM3A, IL36G, AICDA, CREBRF	−2.99	1.900	3.538
GO:0048017	GO Biological Processes	19_GO:0023052 signaling	Inositol lipid‐mediated signaling	CA8, CBL, ENTPD5, CSF1R, GATA3, NKX3‐1, RPS6KB1, SOX9, BTN2A2, FBXL2, KBTBD2, PLEKHA1, DIPK2A	−2.864	2.656	3.723
GO:0044539	GO Biological Processes	19_GO:0051179 localization	Long‐chain fatty acid import	CD36, RPS6KB1, SPX	−2.787	2.014	5.574
GO:1905954	GO Biological Processes	19_GO:0048518 positive regulation of biological process	Positive regulation of lipid localization	CD36, MYB, NKX3‐1, LRAT, IKBKE, EHD1, LDLRAP1	−2.058	3.014	3.113
Reactome gene sets							
R‐HSA‐400206	Reactome Gene Sets	NA	Regulation of lipid metabolism by PPARalpha	ARNTL, CD36, RORA, SP1, MED21, GLIPR1, MED13L, AHRR, CHD9	−2.518	3.028	3.549

## 4 DISCUSSION

Worldwide, the adult population with Down's syndrome is growing, and there is a lack of clinical guidelines for these patients' care (Tsou et al., 2020). DS populations carry a higher risk for chronic degenerative diseases such as cardiovascular illnesses, diabetes mellitus, dyslipidemia, metabolic syndrome, and neurodegenerative diseases such as Alzheimer's dementia (Tsou et al., 2020). Therefore, to improve the preventive and therapeutic approach to DS comorbidities, it is necessary to understand them from their molecular level to their clinical features. The small non‐coding miRNAs play roles at early pathological stages and mobilize through the bloodstream, making their study feasible by non‐invasive methods (Weber et al., 2010). Therefore, the present study analyzed the profile expression of pathological circulating chromosome 21‐encoded miR‐155 and let‐7c miRNAs in DS and euploid populations. These miRNAs have been previously implicated in neurodegeneration and are also linked to dyslipidemias and early‐stage development of Alzheimer's pathogenesis (McGowan et al., 2018; Tili et al., 2018).

First, we characterized clinical features related to metabolism in DS and control populations, as this aneuploidy may affect the profile expression of miRNAs when compared to a euploid control. As expected, we found an increased height for the control group, similar to reported short stature as a typical feature in DS, and growth velocity markedly reduced, mainly between the ages ranging from 6 months to 3 years (Anneren et al., 1993). Moreover, we also observed that BMI was larger in DS than the control in the older group analyzed (B groups), while no differences were observed between the younger groups (A control and A DS groups). Previous studies in children and adolescents revealed that children with DS have a healthy BMI, which may be due to parental control in their diet (Melville et al., 2005). However, during adulthood, DS individuals have greater independence for choosing their food, which tends to have a higher caloric content (Grammatikopoulou et al., 2008). Risk factors associated with the development of obesity such as a decrease in basal metabolism, are also present in DS (Allison et al., 1995; Luke et al., 1994). Moreover, individuals with DS have muscular hypotonia, a phenotype characterized by difficulty in performing activities that involve physical attrition, contributing to a sedentary lifestyle, and, consequently, to weight gain (Korenberg et al., 1994; Shahabi et al., 2017).

In addition to obesity and dyslipidemias, alterations in miRNA expression could synergize the risk for impaired cognition and have a possible link with AD's early age onset. The miRNAs let‐7c and miR‐155, both present on chromosome 21, were selected for their importance in phenotype variability generating haploinsufficiency of target genes and their possible relation with the development of Alzheimer's disease in patients with DS, even at early ages.

**FIGURE 4:**
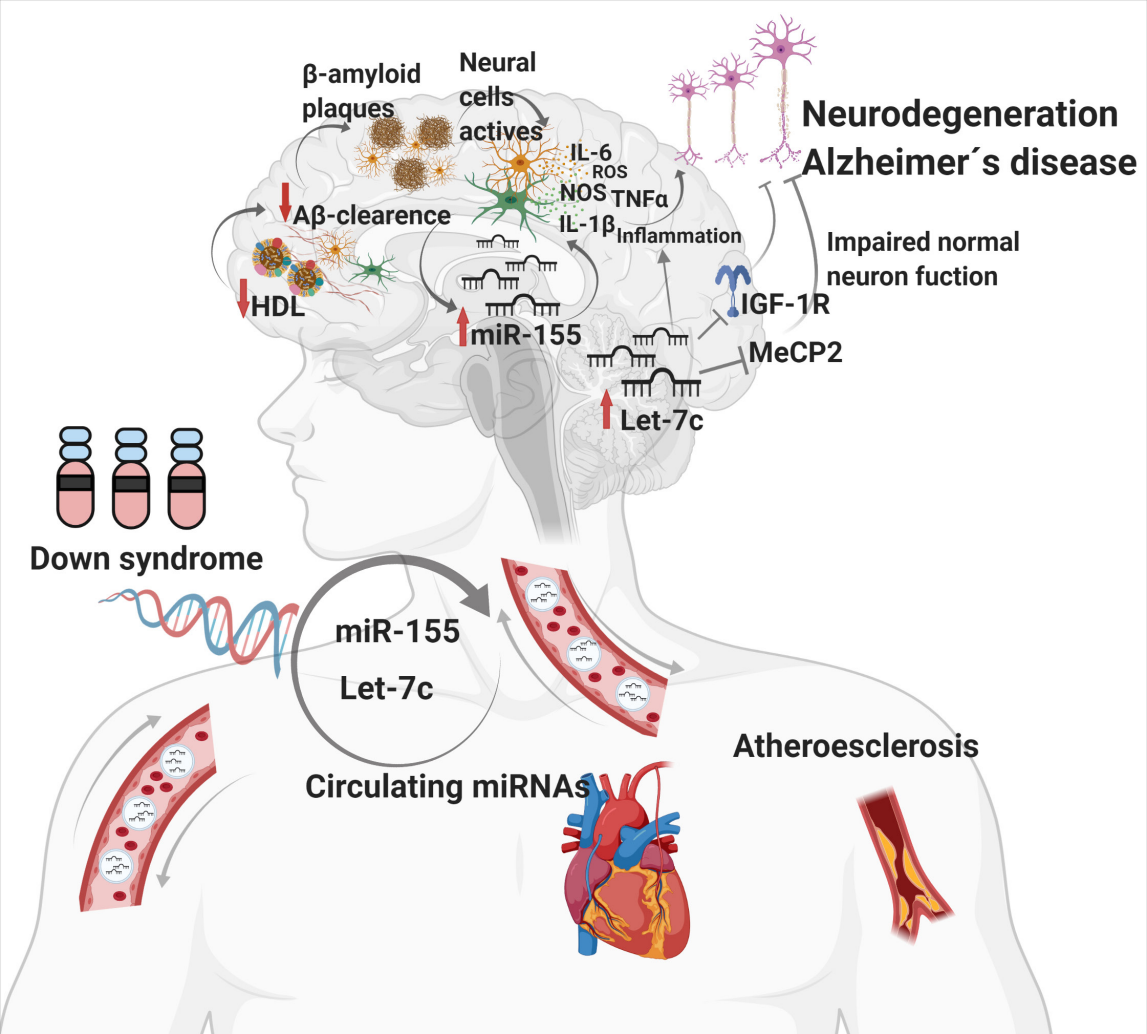
Proposed role of circulating let‐7c and miR‐155 in the Down's syndrome neurodegeneration

Notably, our profiling results highlight a 2.2‐fold expression increase of circulating let‐7c expression in the DS group compared to the euploid population. Following Tili et al. (Tili et al., 2018), who recently reported an upregulation of let‐7c in the DS population using samples from brain tissues (*n* = 6) and observed a high heterogeneity in the cortex (*n* = 4) ranging from 1.63 to 16.48‐fold change (*n* = 3). In a separate study, Xu et al. (2013) also found let‐7c overexpression (1.5‐fold‐change, *n* = 6) in DS lymphocytes. We consider that our study also contributes to a better characterization of the circulating let‐7c that reaches all tissues, including the brain, and its pathophysiological effects may synergize to the DS comorbidities, such as dementia (Figure 4) (Tili et al., 2018; Xu et al., 2013). Based on the results obtained and the previous evidence, to better understand the role that let‐7c and miR‐155 play in the molecular neurodegeneration puzzle, we constructed the drawing shown in Figure 4, reference hereafter thorough discussion.

It is well known that let‐7c is involved in mechanisms related to neurological alterations as an activator of TLR7, in both immune cells and neurons, damaging the CNS through the production of proinflammatory molecules such as the tumor necrosis factor α (TNFα). Moreover, it activates apoptosis via caspases 3 which in turn induces neurodegeneration and neuronal cell death (Figure 4) (Lehmann et al., 2012). Overexpression of let‐7c in neurons also led to morphological and synaptic alterations, marked reduction in neuronal excitability through the inhibition of MeCP2 (McGowan et al., 2018), and decreased expression of the IGF‐1R, which is expressed throughout the brain (Figure 4) (Liu et al., 2018).

Furthermore, IGF‐1R/Insulin alterations are characteristic in patients with AD, Parkinson's, and other neurodegenerative disorders. IGF‐1 is an essential factor for healthy growth and development, involved in neuronal survival, myelin synthesis, cell metabolism, astrocyte function, angiogenesis, neuronal excitability, and oligodendrogenesis (Figure 4) (Bassil et al., 2014). Also, a decrease in IGF‐1 has been reported in people with DS (Anneren et al., 1990; Barreca et al., 1994; Torrado et al., 1991). Moreover, overexpression of let‐7c in the brain of DS adults with dementia was observed, demonstrating its involvement with this process (Tili et al., 2018). Let‐7c overexpression is also related to mitochondrial cardiomyopathies. In trisomic hearts, down‐regulation was observed of the *SLC25A4/ANT1* gene (Solute carrier family 25 member 4/ Adenine nucleotide translocator 1, OMIM #103220), a predicted target of let‐7c and associated with mitochondrial and cardiac anomalies(Izzo et al., 2017). Besides, mitochondrial dysfunction is closely linked to the increase in oxidative stress, which plays a central role in the pathogenesis of DS and the etiology of different intellectual disabilities, as it affects highly oxidative tissues such as the brain, suggesting a synergism between let‐7c and oxidative stress (Esbensen, 2010; Perluigi & Butterfield, 2012; Valenti et al., 2014; Wallace & Fan, 2010).

Regarding circulating miR‐155 expression, we do not observe significance in the DS people studied. Previous studies using post‐mortem brain tissue of 34‐42‐year‐old donors with DS and dementia found a significant up‐regulation of this miRNA (Tili et al., 2018). Interestingly, in the DS mouse model Ts65Dn, miR‐155 showed significant over‐expression in the hippocampus and whole blood, but not in lung tissue. Also, there was a down‐regulation of the mRNA target *Ship1*(inositol phosphatase) in the Ts65Dn hippocampus, but not in lung (Bofill‐De Ros et al., 2015). The previous evidence and our results suggest that physiopathological changes in miR‐155 expression may be tissue and temporally specific. In‐depth follow‐up studies are necessary to analyze these changes in the brain tissue to understand its contribution to neurodegeneration in DS.

The role in the development and adult central nervous system (CNS) of several miR‐155 gene targets has been demonstrated in murine and cellular models (Keck‐Wherley et al., 2011; Tili et al., 2018). miR‐155 predicted gene target pathways are highly enriched for adhesion molecules such as Pcdhs, which are type I transmembrane receptors expressed predominantly in the central nervous system (CNS) and located in part in synapses involved in pivotal developmental processes, such as axon guidance and dendrite arborization. Changes in their expression might play a role in DS, as has been demonstrated for other neurodevelopmental disorders such as autism, encephalopathy epilepsy, Fragile X syndrome, and neurodegenerative diseases (Keeler et al., 2015; Kim et al., 2015; Yan et al., 2014).

Notably, our results showed a negative correlation between miR‐155 expression and HDL‐cholesterol levels. miR‐155 is a multi‐functional miRNA implicated in several pathological processes such as hematopoietic lineage differentiation, immunity, inflammation, cardiovascular function, and neurodegeneration. It has a significant role as a component of inflammatory signal transduction in the pathogenesis of atherosclerosis due to its expression in endothelial cells, macrophages, dendritic cells, vascular smooth muscle cells (VSMCs), and differentiation of leukocyte subsets (Ma et al., 2013). Human in vivo studies have shown that miR‐155 is up‐regulated in atherosclerotic lesions while, its circulating levels are reduced (Ma et al., 2013). Also, animal models have shown enhanced atherosclerosis in LDL‐R^−/−^ mice (Donners et al., 2012) and reduced atherosclerosis in Apoe^−/−^ mice (Kuziel et al., 2003). It was additionally found that miR‐155 targets LDL receptors, down‐regulating lipid uptake. These findings suggest that up‐regulation of miR‐155 contributes to high circulating LDL levels. Nevertheless, there is no direct evidence for mechanisms that relate miR‐155 expression with HDL levels. HDL is the only form of cholesterol in the brain, is rich in the apolipoprotein ApoE, and contributes to Aβ peptide clearance. ApoE is present in circulating LDL‐C and functions as its ligand for cellular lipid uptake. There are three isoforms for ApoE, ApoE2‐4, from which ApoE4 is associated with higher serum levels of triglycerides and LDL‐C contributing to atherosclerosis. Nevertheless, in the brain, ApoE4‐HDL is less effective in the clearance of Aβ peptide thanApoE2/3‐HDL (Kuziel et al., 2003).

To understand the mechanism that links miR‐155 mRNA targets to HDL‐C levels, we performed a deeper in silico analysis, which showed that it might modulate molecules such as the aryl hydrocarbon receptor nuclear translocator like (*ARNTL*, OMIM #602550), also known as Brain and Muscle ARNT‐Like 1(*BMAL1*, OMIM #602550). *ARNTL* encodes a BHLH transcription factor, which plays a role in the cellular response to lipid, fat cell differentiation, and the pathway of the regulation of lipid metabolism by PPARalpha (Table 4). Lee et al., 2012 showed that the CLOCK/BMAL1 complex act up‐regulating human LDLR promoter activity, suggesting a role for this gene in cholesterol homeostasis (Lee et al., 2012). Moreover, this gene is also responsible for the regulation of glucose homeostasis and metabolism, which can lead to hypo‐insulinemia or diabetes when disrupted (Marcheva et al., 2010). Altogether, these observations reinforce the possible link of miR‐155 targets with lipid metabolism observed in DS patients (Figure 4).

Down syndrome associated‐neurodegeneration such as Alzheimer's disease, is a multifactorial puzzle linked to an altered genetic background, including alterations in miRNA expression, but potentiated by dyslipidemias (Mahley, 2016; Real de Asua et al., 2014; Tokuda et al., 2000). Elevated cholesterol levels induce accelerated APP processing in its amyloidogenic pathway, a condition related to the development of Alzheimer's dementia (Bodovitz & Klein, 1996; Burns et al., 2003; Buxbaum et al., 2001; Simons et al., 1998). Studies of the lipid profile in children with DS, compared with their euploid siblings, adjusting for BMI showed that even in normal body weight, the DS children presented alterations in cholesterol, triglycerides, HDL, and LDL (Adelekan et al., 2012; de la Piedra et al., 2017). Our results demonstrate that individuals with DS have alterations in the lipid profile compared to controls at an early age as an increase in cholesterol, triglycerides, and low levels of HDL; and consistent in adults with DS. These results support the idea that the alterations in lipid metabolism are not only due to obesity but possibly to the presence of an extra copy of chromosome 21, thus constituting a risk factor for AD neurodegeneration. Several reports have determined a positive correlation of Aβ levels with elevated cholesterol and low HDL levels (Ehehalt et al., 2003; Tokuda et al., 2000; Wahrle et al., 2004). HDL maintains the transport of cholesterol to neurons for functioning and has a role in Aβ clearance; hence, high cholesterol levels and low HDL levels are related to cognitive disorders (Figure 4) (Ehehalt et al., 2003; He et al., 2016; Tokuda et al., 2000; Wahrle et al., 2004). In line with this evidence, we found that let‐7c overexpression was higher among DS individuals with low‐HDL levels. Still, we did not find significative correlation between let‐7c and HDL or mRNA targets related to lipid metabolism by *in‐silico* analysis. Since HDL particles transport miRNAs (Baldan & de Aguiar Vallim, 2016), it may be possible that HDL plays a role in proinflammatory let‐7c clearance altogether amyloid peptides elimination. Also, we found that miR‐155 levels negatively correlate to low HDL levels in the DS population. Even though no mRNA targets for miR‐155 involved in lipid metabolism have been demonstrated, based on our *in‐silico* analysis, a direct relationship between its expression and lipid profile alterations is likely. However, the fact that there is a negative correlation of miR‐155 expression with HDL level or a differential expression of let‐7c according to lipid levels suggests the existence of a complex molecular network linking both parameters.

Overall, our results suggest that a set of differentially expressed miRNAs promotes the progression of cognitive impairment in patients with DS by regulating genes of biological pathways involved in lipid metabolism and nervous system development. Moreover, other biological processes such as inflammation and the immune system must integrate for Alzheimer's neurodegeneration in the DS puzzle (Figure 4).

## 5 CONCLUSION

Our results reveal an up‐regulated expression pattern for circulating let‐7c miRNA and an inverse correlation between miR‐155 expression and low HDL‐C levels. The data suggests that in Down syndrome, the chromosome 21‐encoded let‐7c and miR‐155 may be dysregulated and, together with dyslipidemia, may play a role in the homeostasis of molecules related to cognitive function and early development of Alzheimer's disease dementia. This implies that modulating the expression of these miRNAs in the CNS may help abrogate the inevitable pathway to dementia in adults with Down syndrome.

### ETHICAL COMPLIANCE

The study followed the Declaration of Helsinki and Good Clinical Practice guidelines provisions and was approved by the Research Ethics Committee at the Medical School of the Autonomous University of Sinaloa, which has a national certification (CONBIOÉTICA‐25‐CEI‐003‐20181012). Parents or legal guardians of people with DS were informed before the evaluation and provided written consent to undergo study procedures.

## CONFLICT OF INTEREST

The author declares no conflict of interest.

## AUTHOR CONTRIBUTIONS

Pérez‐Villarreal JM and Aviña‐Padilla K contributed to the study design, performing experiments, collection, analysis and interpretation of data, and drafting the paper. Beltrán‐López E, Guadrón‐Llanos AM, and López‐Bayghen E assisted with technical support, patient data collection, ideas, and critical analysis, and interpretation of data. Meraz‐Ríos MA and Varela‐Echevarria A contributed to the writing, reviewing, and discussion of the manuscript. Magaña‐Gómez J and Angulo‐Rojo C made a substantial contribution to conception and design of the project, analysis and interpretation of data, and writing and revising the paper to the final version to be published. Angulo‐Rojo C was responsible for the funding support and the general supervision of the research.

## Supporting information


Figure S1

Figure S2

Appendix S1

## Data Availability

Data are available on request from the authors.
