# Supplementary figures and images for: Profiling of circulating chromosome 21‐encoded microRNAs, miR‐155, and let‐7c, in down syndrome
Source: Mol Genet Genomic Med. 2022 Apr 12;10(6):e1938. doi: 10.1002/mgg3.1938 (PMC9184673; doi:10.1002/mgg3.1938)

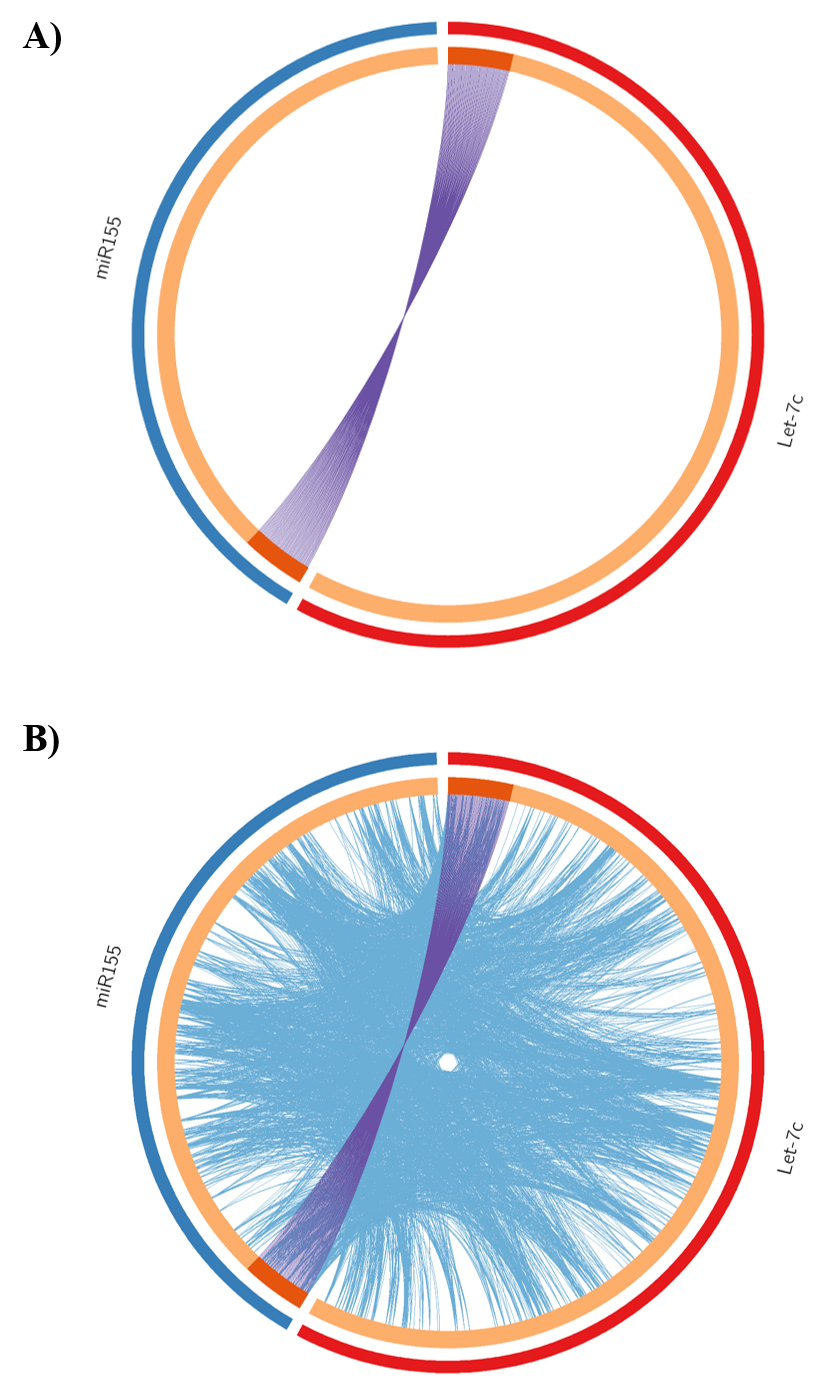

Supplement: Supplementary file 1 — Figure S1 [file MGG3-10-e1938-s001.tif]

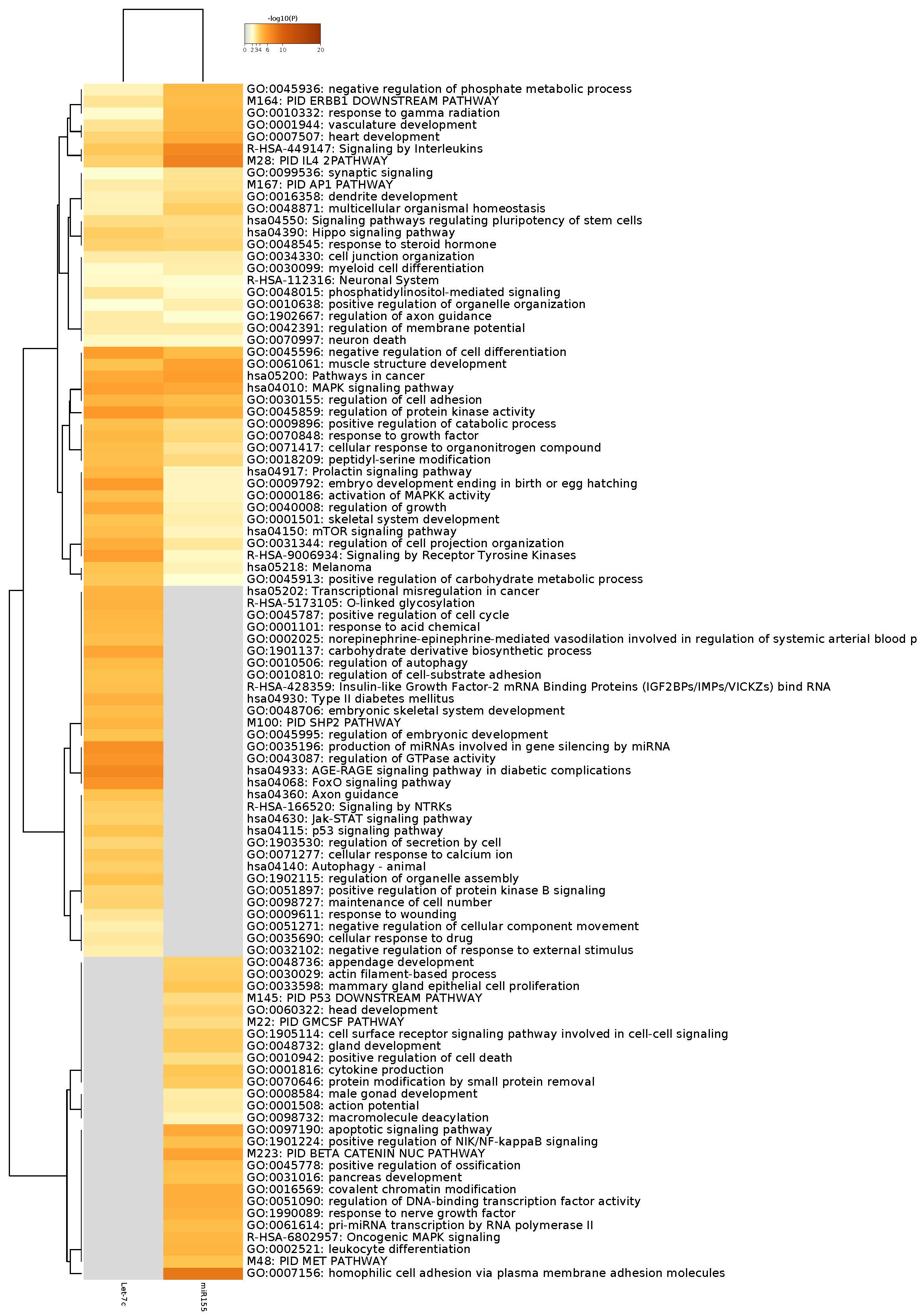

Supplement: Supplementary file 2 — Figure S2 [file MGG3-10-e1938-s003.jpg]
