# Supplementary material for: Profiling of circulating chromosome 21‐encoded microRNAs, miR‐155, and let‐7c, in down syndrome
Source: Mol Genet Genomic Med. 2022 Apr 12;10(6):e1938. doi: 10.1002/mgg3.1938 (PMC9184673; doi:10.1002/mgg3.1938)
Supplement: Supplementary file 3 — Appendix S1 [file MGG3-10-e1938-s002.docx]

**Supplementary figures captions**

**S1 Fig.** **Circus graphs of overlapping and divergent targets genes of miRNA 155 and Let-7c.** A) only at the gene level, purple curves link identical genes; B) including the shared term level, blue curves connect genes that belong to the same enriched ontology term between the targets of both miRNAs. The inner-circle represents gene lists, where hits are arranged along the arc. Genes that hit multiple lists are colored in dark orange, and genes unique to a list are shown in light orange.

**S2 Fig. Networks of enriched ontology clusters for miR-155 miRNA regulated targets.** 100 GO: BP, Gene Ontology: Biological Processes terms.
